# Enhancing diversity in language based models for single-step retrosynthesis

**DOI:** 10.1039/d2dd00110a

**Published:** 2023-02-16

**Authors:** Alessandra Toniato, Alain C. Vaucher, Philippe Schwaller, Teodoro Laino

**Affiliations:** a IBM Research Europe Saümerstrasse 4 8803 Rüschlikon Switzerland ato@zurich.ibm.com; b National Center for Competence in Research-Catalysis (NCCR-Catalysis) Zurich Switzerland

## Abstract

Over the past four years, several research groups demonstrated the combination of domain-specific language representation with recent NLP architectures to accelerate innovation in a wide range of scientific fields. Chemistry is a great example. Among the various chemical challenges addressed with language models, retrosynthesis demonstrates some of the most distinctive successes and limitations. Single-step retrosynthesis, the task of identifying reactions able to decompose a complex molecule into simpler structures, can be cast as a translation problem, in which a text-based representation of the target molecule is converted into a sequence of possible precursors. A common issue is a lack of diversity in the proposed disconnection strategies. The suggested precursors typically fall in the same reaction family, which limits the exploration of the chemical space. We present a retrosynthesis Transformer model that increases the diversity of the predictions by prepending a classification token to the language representation of the target molecule. At inference, the use of these prompt tokens allows us to steer the model towards different kinds of disconnection strategies. We show that the diversity of the predictions improves consistently, which enables recursive synthesis tools to circumvent dead ends and consequently, suggests synthesis pathways for more complex molecules.

## Abstract 2

Current Al solutions to chemical retrosynthesis focus on predicting the reported ground truth, not taking into account the ability to generate alternatives. Our work is the first Al approach tackling and analysing retrosynthetic diversity directly.

## Graphical abstract

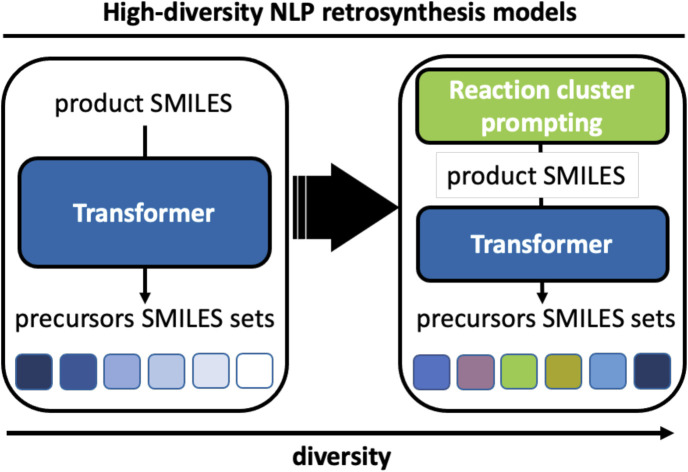

## 1 Introduction

Finding the optimal combination of readily available chemical building blocks to produce a desired molecule is the Holy Grail of synthetic chemistry. The objective is to infer the individual (reaction) steps leading to a target material from known starting materials. This method, known as retrosynthesis, is a technique that was long thought to be the exclusive domain of a small but dedicated group of experts. In today's world, retrosynthesis is crucial to solving many materials problems. Still, a growing number of experts are challenged by the complexity of the vast corpus of publicly available chemical information. Computers lead to the development of rule-based algorithms in which disconnection rules were applied to appropriate molecules to achieve the desired transformation. Recent research has leveraged the powerful Deep Learning models to solve the problem and automate the operation while still allowing for the skilled oversight of human chemists. Different models have been proposed^1–12^ and are usually classified as template-based, semi-template and template-free models. The template-based models, pioneered by Segler and Waller,^1^ are trained to predict pre-extracted rules, while the semi-template models generate reactants from a product, first identifying intermediate molecules, and, second, completing these into reactants by sequential generation of atoms. This is the case of the work of Somnath *et al.*^9^ On the other hand, the template-free approaches learn the retrosynthetic rules from the training data. Translation, in particular, is one of the established ways of doing retrosynthesis with Machine Learning. Liu *et al.*^3^ have pioneered the field introducing sequence-to-sequence methods, while Schwaller *et al.*^6^ proposed transformers for reaction prediction and retrosynthesis. After them, several models leveraging the potential of augmentation and pretraining (BERT-like models) have been developed for retrosynthesis or related chemistry tasks. For instance, Tetko *et al.*^12^ looked at augmentation, while Pesciullesi *et al.*^13^ studied transfer learning.

**Fig. 1:**
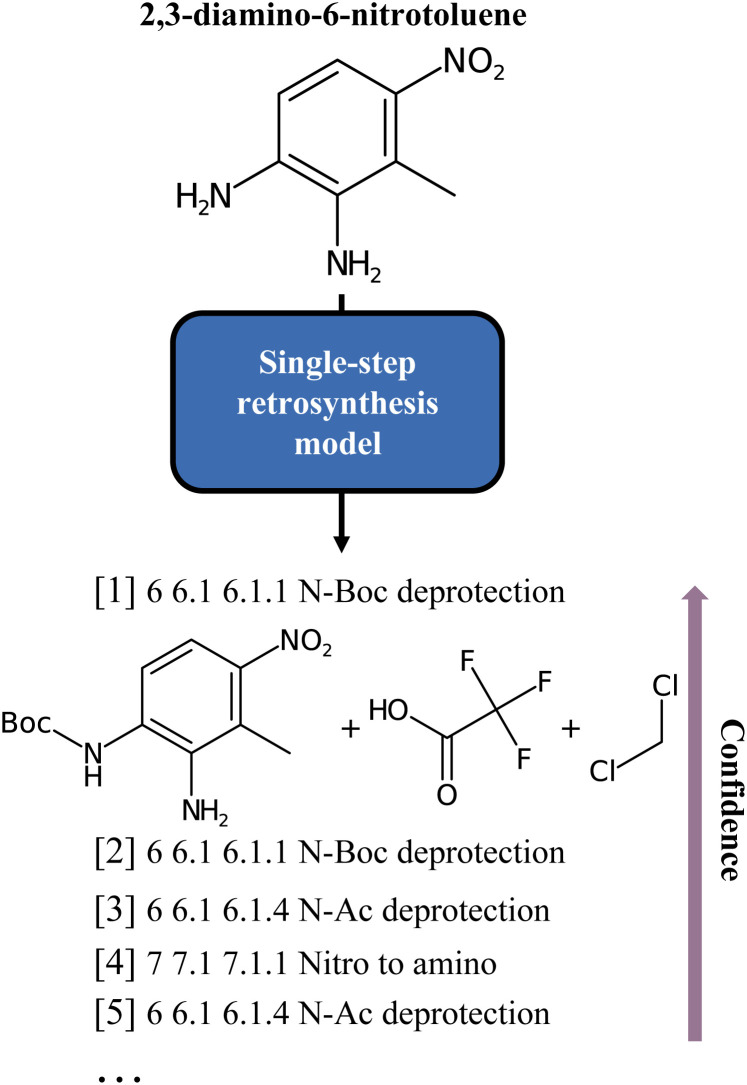
Classes of the single-step baseline predictions for the 2,3-diamino-6-nitrotoluene molecule, as produced by Schwaller *et al.*^6^ As can be noted, all but one are different forms of deprotection ordered by model confidence.

Irrespective of the approach, either template-free or template-based, the principle that underlies all these methods is the same: a model is trained on some data (often the compound to synthesize, given as a text string, an embedding, or a graph) and then evaluated by comparing its output to a target (the set of “optimal” precursors). However, this perspective is sometimes at odds with the chemistry at hand. In fact, for each target molecule, there is generally a wide variety of valid disconnections that connect the target molecule to different sets of precursors. If the dataset were hypothetically perfectly balanced, all conceivable reactions leading to a target molecule would be evenly represented, but in practice this is far from being the case. Existing reaction datasets, and consequently models, give more weight to well-represented reaction classes, thus penalising more interesting but less frequent disconnections. For example, Fig. 1 shows an insufficient diversity for the proposed list of disconnections. Here, we interpret diversity as “chemical class diversity”, considering a model more diverse in its predictions if these belong to different reaction classes as defined by NameRXN.^14^

To increase the diversity of the predictions in single-step text-based retrosynthesis models and counteract the effect of imbalanced datasets, we propose a prompt-based scheme to enhance and guide more diversity in the language model predictions. We introduce a modified transformer-based model.^6,15^ Inspired by works in natural language processing for prompt-based learning,^16–19^ we show that concatenating a class information during training (as an additional token), leads to more diverse predictions at inference. We experiment with different classification strategies, including clustering reaction fingerprints^20^ to evaluate the adequate number of tokens. We compare the cluster token prompt model to a baseline translation model in terms of top*n* accuracy, round-trip accuracy, class diversity and coverage. After training our model on the proprietary Pistachio^21^ data, we increased the class diversity of the predictions to an average of 5.3 for each reaction target compared to 1.9 of the pristine model, while retaining a high value of 62% for the round-trip accuracy of the disconnections.

## 2 Results and discussions

### 2.1 Introducing the cluster token prompt

**Fig. 2:**
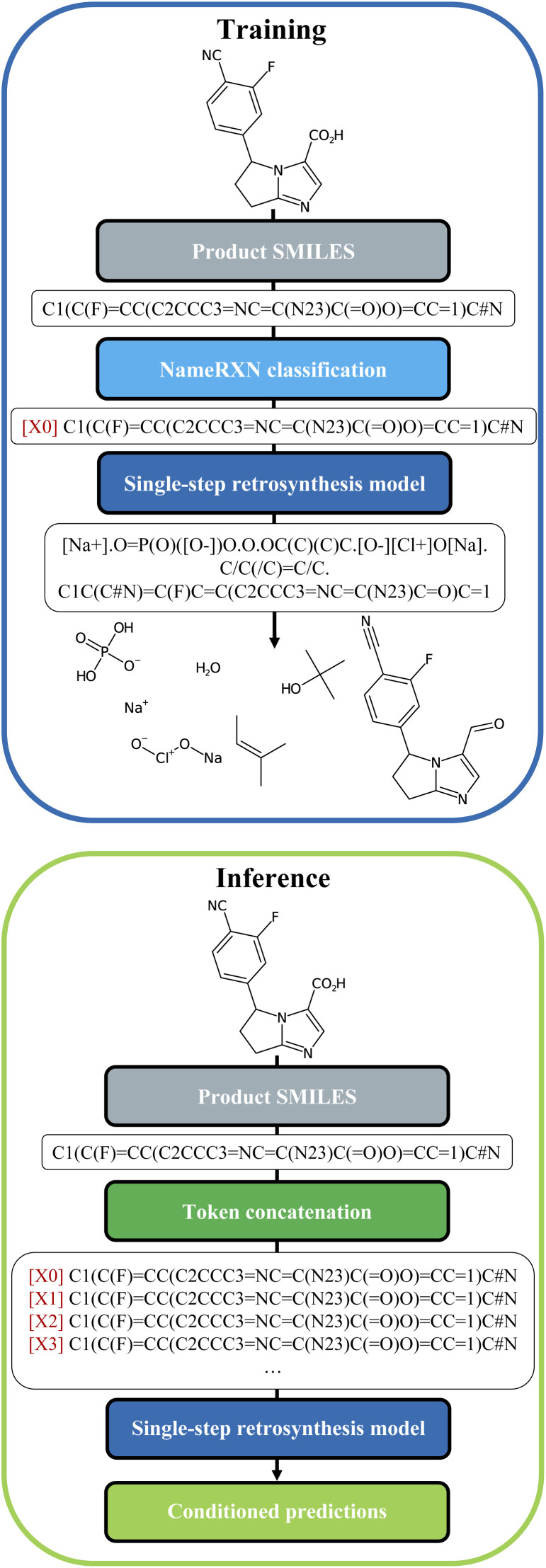
Top: Data-preprocessing procedure for training. The cluster token is prepended to the product SMILES of the reaction and the set of precursors is used as the target. Bottom: Data-preprocessing procedure for inference. A new string is generated for each molecule where each different available cluster token is concatenated. Conditioned predictions are then collected for each molecule.

We built our one-step retrosynthesis model out of the Transformer model.^6,12,15^ Transformer models learn a representation of each token in the input string. To represent molecules, we use the simplified molecular-input line-entry system (SMILES) language,^22,23^ where atoms and bonds are codified as specific combinations of text characters. Schwaller *et al.*^24^ developed the tokenization regex used to tokenize the SMILES. Examples of SMILES strings can be found in Fig. 2. The embeddings learned for each token depend on the context, which allows the model to encode much more subtle information than a pure one-hot encoding of an atom or bond. To increase diversity, we prepended during training a new token, corresponding to the cluster, or class, to which the reaction belongs. The cluster is indicated in front of each input SMILES product molecule. The clusters were defined in two different ways: first, using the NameRXN classification schema, and second, using a *k*-means clustering algorithm on top of reaction fingerprints^20^ (more details in Section 3.3). The NameRXN classification provides a class labelling schema where the top level denotes the superclass, the middle level denotes the reaction category, and the final level is the named reaction classification.^14^ This naming schema is based on the RXNO ontology.^25^ To create the prompts, only the top-level indices were used: 0 → unrecognized, 1 → heteroatom alkylation and arylation, 2 → acylation and related processes, 3 → C–C bond formation, 4 → heterocycle formation, 5 → protections, 6 → deprotections, 7 → reductions, 8 → oxidations, 9 → FGI, 10 → FGA, 11 → resolutions. The data-preprocessing procedure for training can be visualized in Fig. 2 (top).

At test time, the input product molecule can be concatenated to all the available cluster tokens (see Fig. 2, bottom), generating *X* equivalent inputs, where *X* is the number of cluster tokens used. The first token seen by the transformer is the cluster token. This will steer the predictions towards typical disconnections for that class. Collecting all the predictions that were ranked the highest by the model, commonly called top one (top1) predictions, for the *X* class-tokens (and possibly additional predictions for each of the *X* class-tokens), leads to a set of disconnections more diverse than the top*N* outputs of a regular Transformer model, which we use as a baseline. The advantage of this strategy is that the steering acts as a weak influencer of the predictions, rather than a forcing term, such as using a certain template, which can either lead to failure or success. In comparison to the baseline model, the cluster token prompt approach allows the model to “select” from a limited pool of options while yet leaving it with much flexibility. In the following section, we present our models and the results in more details.

### 2.2 High diversity single-step retrosynthesis models

As a training corpus, we utilized the proprietary reaction dataset Pistachio,^21^ consisting of 2 4 47 596 unique reactions with both precursors and products in SMILES format. In addition, we tested the procedure on the public dataset USPTO 50k,^26^ processed by Ramsundar *et al.*,^27^ which we provide together with the code (available as additional material). Results for this dataset can be found in Appendix A.

The data were first suitably pre-processed (see Section 3.1). We used two ways to produce the cluster tokens to prepend in front of each reaction: the first one relies on the NameRXN classification and the second one on a K-means clustering algorithm. For the K-means clustering, we identified the clusters with the reaction fingerprints^20^ (see Section 3.3 for details). The models tested are described below:

• baseline: a Transformer model^6,15^ with no cluster-token information.

• 12clusters: a model that utilizes as tokens all the first level classification available from NameRXN (*i.e.* classes from ‘0’ to ‘11’).

• 3clustersRandom: a model built on top of the 12 classes from NameRXN which we grouped randomly in 3 clusters.

• 4clustersRandom: same as the model above, but with 4 clusters.

• 3clustersKmeans: this model results from the application of the K-means clustering algorithm with 3 clusters on the 3 dimensions obtained from a PCA analysis of the reaction fingerprints.

• 4clustersKmeans: same as the model above, but with 4 clusters.

• optimalKmeans: in this model, we estimated the optimal PCA dimension for the fingerprints (14) and the optimal number of clusters (10). The procedure is described in Section 3.3.

Once the token was identified for each reaction, it was prepended to the SMILES string with the following format: [*i*] for *i* = 0…*X* (see Fig. 2), with *X* being the number of tokens available in each of the models.

For the models evaluation, we split randomly the data into a training/validation/test set with a proportion of 80/10/10 for five different random seeds, and we proceeded as follows:

(1) We chose one of the splits randomly and we trained all the cluster token prompt models. We tested them against the validation set and chose the best performing model.

(2) Then, we merged the train and validation set for the five different seeds and trained the best prompt-based model plus the baseline model.

(3) We compared the so-trained baseline and best models against the test sets.

Each of the trained models, including the baseline, was trained for 260 000 steps with 1 GPU (approximately 48 hours of training). Indeed, at later checkpoints no improvement over the loss function was observed.

**Fig. 3:**
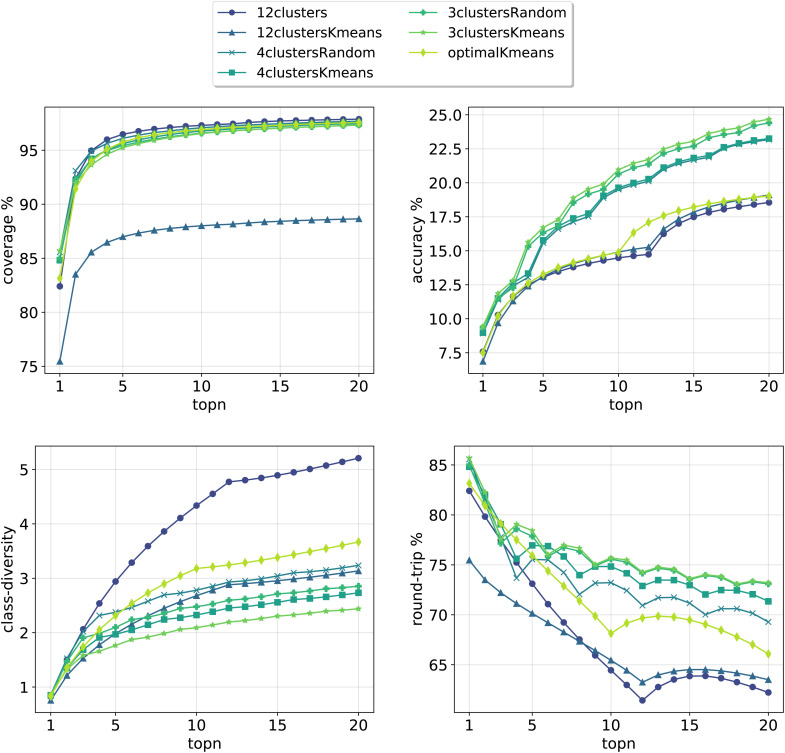
Model metrics. Top left: coverage. Top right: top*n* accuracy. Bottom left: class diversity. Bottom right: round-trip accuracy. For the definition of each metric please refer to Section 3.4.

In Fig. 3, we report the results for the prompt-based models evaluated on the validation set. For each model, we retained the top24 predictions as X*top*k* = 24 = top*N* where *X* is the number of class tokens for each model and top*k* is the number of predictions retained for each token-concatenated sample (*e.g.* for the 12clusters model, *X* = 12 and top*k* = 2). The plots report 4 metrics of interest as a function of the number of top*N* predictions analyzed (see Section 3.4 for the metrics definition). To properly compare models, we looked only at top20 predictions (and not top24), as for the optimalKmeans model only 20 predictions per sample were produced (2 for each token-conditioned input).

All cluster token models show a good coverage (above 95%) after top3 predictions. The 12clustersKmeans model is the only one performing poorly from this point of view. Looking at the accuracy, we see that it increases slowly and reaches a top20 value between 18% and 25% for all models. In addition to reactants, our retrosynthesis models predicts a wide range of precursors, and is not limited to the disconnected fragments only. Therefore, many times the ground truth appears with a slightly different set of reagents, justifying the low accuracy values. Accordingly, when a model can produce multiple correct answers, accuracy is not the most crucial metric to consider. Different publications have been questioning the suitability of the top1/top*N* accuracy for single-step retrosynthesis models.^6,28^ We consider the value of the round-trip accuracy to be more interesting (see Section 3.4). This value measures the ability to recover the input molecule by running a forward reaction model on top of the predicted precursors (details on the forward model are in Section 3.2). This metric decreases with the number of top*N* predictions considered. The decay is more consistent for models utilizing a greater number of tokens (12clusters, 12clustersKmeans). Note that this is to be expected, since we are asking for disconnection conditions that may be impossible to satisfy for some input molecules. However, a high value of coverage guarantees at least one proposed valid disconnection per input molecule. It is important to note that round-trip accuracy does not take into consideration that the top20 predictions for a sample, even if correct, can all collapse into one. This happens for example if the model predicts an identical set of reactants multiple times (or for the case with reagents, multiple times the same reactants and a different solvent). For this reason, the final metric that we report, the class diversity, is the most interesting one as it takes into account all these challenges. It measures the average of the different (NameRXN) classes predicted for a given input, considering only the valid predictions (see Section 3.4). The value highly depends on the number of cluster tokens used and differs from one strategy (NameRXN) to the other (K-means clustering). As a clarifying example, a class diversity of 5 means that there are at least 5 valid predictions that are fairly different. A baseline with an average class diversity of 1.9 for 20 predictions means that even if all predictions are valid, on average only 1.9 are interesting because of being distinctly different from one another.

Using more tokens results in more diversity in the predictions (5.2 for the 12clusters model at top20 predictions), but also a higher number of incorrect predictions. The 12clustersKmeans model instead loses in round-trip accuracy without a relevant compensation on the class diversity side. The most interesting models are the 12clusters, from the point of view of the increased class diversity, and the optimalKmeans, which reaches decent values of class diversity and could be used also in a setting where the reaction classification labels are not available.

**Fig. 4:**
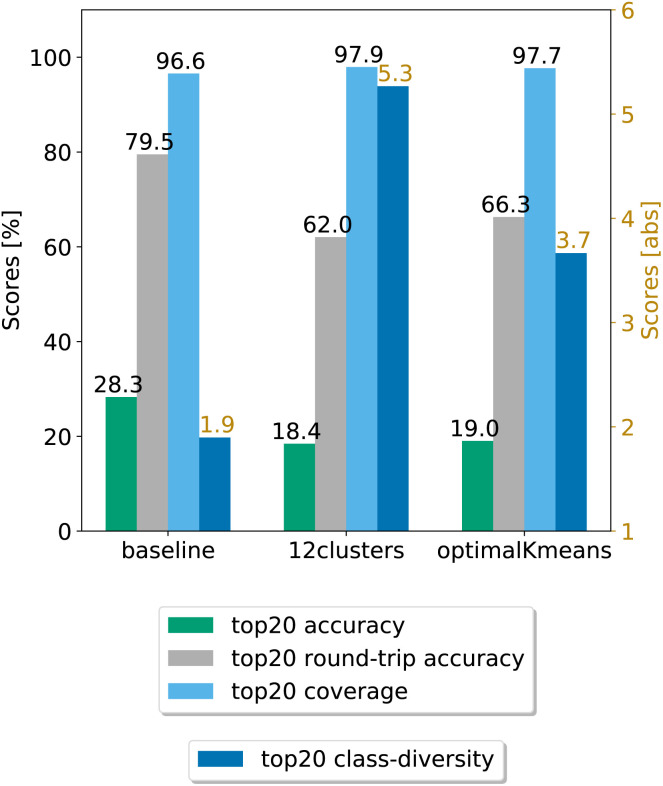
Final comparison of the best prompt-based models and the baseline against the test set. The values of the metrics reported are averaged across five random seeds. For convenience, standard error values are reported in Table 1.

In a second step, we chose the best models (12clusters and optimalKmeans), and compared their performance against the baseline. We evaluated our models on five randomly chosen test splits, where, this time, the validation set was included in the training. The results on the top20 predictions are reported in Fig. 4. As can be seen, the prompt-based model does indeed boost the diversity of the predictions. On the test set, we achieve an average boost of class diversity of about 3.4 points for the 12clusters model. For completeness, we report in Appendix B the behaviour of the baseline model and the best models as a function of the top*n* predictions, with standard errors.

Table 1 shows the (top20) metrics with standard error bounds for the three models under consideration, generated from the five different random seed experiments.

**Table 1:** Comparison of the prompt-based models against the baseline on the test set. Uncertainty bounds are computed based on the standard error and reported in the table

Model	Coverage	Accuracy	Round-trip accuracy	Class diversity
Baseline	96.58 ± 0.06%	**28.28 ± 0.05%**	**79.50 ± 0.68%**	1.90 ± 0.01
optimalKmeans	97.69 ± 0.04%	19.02 ± 0.47%	66.27 ± 0.95%	3.67 ± 0.02
12clustersKmeans	**97.94 ± 0.06%**	18.42 ± 0.31%	62.03 ± 0.53%	**5.27 ± 0.05**

**Fig. 5:**
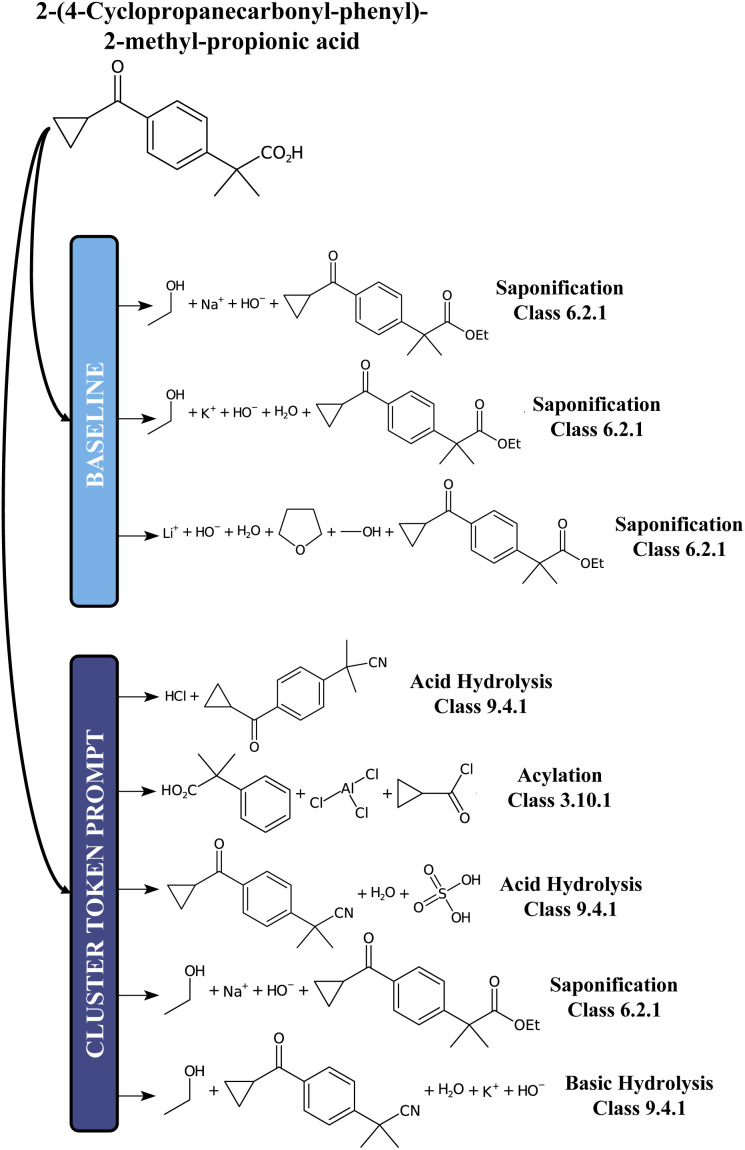
A chemical example predictions with the baseline retrosynthesis model and the prompt-based model.

For comparison, we report in Fig. 5 an example of prediction with the baseline model and the 12clusters model. While for the baseline the proposed disconnections all belong to the class of Saponification reactions (6), for the 12clusters model we observe much more diversity in terms of reaction classes. Also, looking at the main reactants generated, the prompt-based model proposes different alternatives (*e.g.* Acylation reaction *versus* Saponification).

## 3 Methods

### 3.1 Data

For the assessment of model performance we used two datasets derived by text-mining chemical reactions in US patents: the proprietary Pistachio,^21^ and the open-source USPTO 50k.^26^ All reactions in both datasets went through a cleaning procedure, outlined below (the RDKit library was used^29^):

• removal of duplicates and invalid reactions

• merge reactants and reagents: in chemistry reactants are the main actors in the reaction, but they are helped by other molecules that allow the reaction to take place (*e.g.* solvents) even if not contributing atoms to the final product. In our work we merged reactants and reagents (also known as ‘precursors’) on the left hand side of the reaction (*e.g.* A > B > C →   A B ≫ C).

• set on the precursors: given no real relationship between the number of times a molecule appears in the patent reaction and the stoichiometry, we made molecules unique.

• removal of multi-products reactions: this operation was performed after removing residual precursors molecules from the product side.

• removal of reactions where the product contains atom types not present in the precursors side.

• removal of single-atom products.

• removal of reactions where the absolute formal charge exceeded the value of 2.

• removal of reactions where the maximum number of tokens was above 500.

• removal of reactions with the same set of precursors, but different products.

We provide the already cleaned public dataset USPTO 50k^26^ together with the code.

The cleaned dataset was randomly split into training, test and validation sets (80%/10%/10%) for five different random seeds. One of these splits was used to choose the best cluster token model, while the comparison to the baseline was performed against all five random seeds, merging validation and train set.

### 3.2 Models

Our Deep Learning approach to single-step retrosynthesis does not rely on reaction templates and takes into consideration both reactant and reagents as the target set. Predicting all the precursors at once has been common practice in several groups for a long time, especially when language-based models are used. This allows models to support without special attention reactions where the reactant–reagent distinction is subtle; in addition, reagents are, from a chemistry perspective, useful for the general understanding of the mechanism. We formulate the problem of going from the product to the target precursors as a machine translation task, similar to Schwaller *et al.*^6^ The molecules were codified as SMILES strings, tokenized, and fed to the Transformer model.^15^ We used the OpenNMT framework^30^ and PyTorch^31^ to build the models. The hyperparameters were the same used in related work^6,32^ and were kept fixed throughout all simulations. The transformer is made up of a set of encoder layers and a set of decoder layers. The tokens of the input SMILES string are encoded into (learned) hidden vectors by the encoder. Those vectors are then fed to the decoder to predict the output sequence, one token at a time. The model size and hyperparameters where taken from previous literature.^24^ The number of layers in both the encoder and decoder was set to 4 (size 384). The main characteristic of the transformer is the presence of multi-head attention and the number of these heads was set to 8. Dropout was also included in the model at a rate of 0.1. An Adam optimizer was used for loss minimization and the starting learning rate was set to 2. An exhaustive file with all the parameter values used can be found in the code.

#### 3.2.1 Forward and classification models

To better evaluate the single-step retrosynthesis models, two additional models are necessary. The first model is the forward prediction model used for reaction prediction (from precursors to product). This model was built with the same dataset used for the retrosynthesis one, switching source and target. Training files are available together with the code. The second model is a classification model to classify the retro predictions. For this, we also relied on transformers. The procedure is the one of Schwaller *et al.*,^20^ model ‘Transformer enc4-dec1’, applied to the same reaction dataset as the retro and forward model.

### 3.3 K-means analysis

**Fig. 6:**
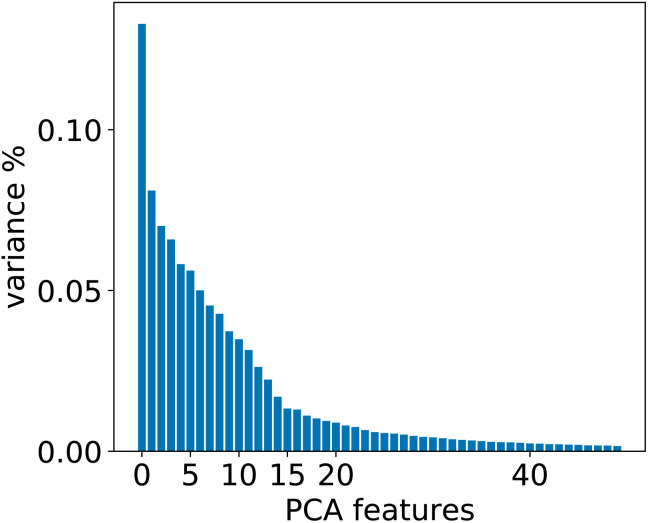
Relevant PCA components analysis. Two drops in the variance are observed around the 2nd/3rd component and a smaller one around the 14th component.

To evaluate whether adequately conditioned predictions can be obtained without relying on *ad hoc* classification, we generated the conditioning tokens starting from reaction fingerprints^20^ and applied a K-means clustering algorithm. Since the fingerprints live in a high-dimensional space, we first reduced their dimension with principal component analysis (PCA). To choose the best number of components we performed a variance analysis and identified the components which capture the greatest amount of variance in the data (see Fig. 6).

For the 12clustersKmeans, 3clustersKmeans and 4clustersKmeans models, we kept only the first three components. For the optimalKmeans model we shot further and included all the first 14 components. Subsequently, for the K-means clustering, we relied on a fixed number of clusters for the first models (12clustersKmeans, 3clustersKmeans and 4clustersKmeans). On the other hand, for the optimalKmeans model, we first performed an analysis to determine the optimal grouping.^33^ This can be done by measuring the sum of the squared distances to the nearest cluster center (inertia). This allows computing a plot of the inertias against the number of clusters used. The optimal *k* is said to coincide with the elbow of the plot, where the inertia value change starts to be less significant. The inertia plots can be found in Appendix C.

**Fig. 7:**
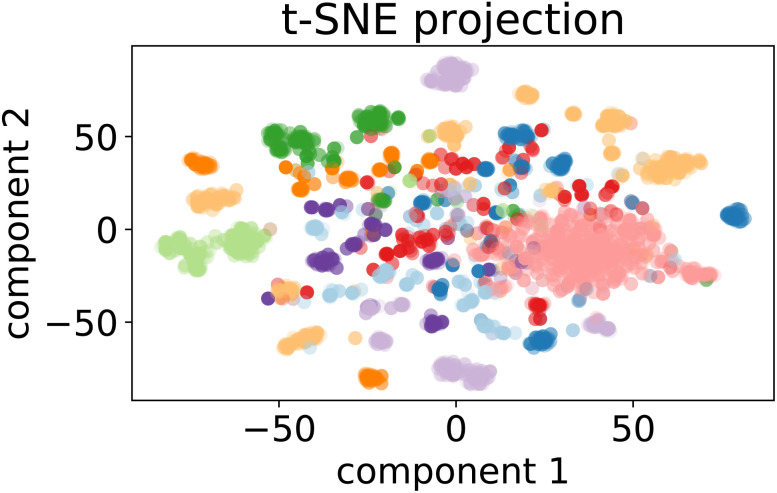
t-SNE projection for 50 000 training samples of the optimalKmeans model. The different colours represent the different K-means clusters.

Fig. 7 shows the clusters generated for the training set of the optimalKmeans model. The plots for the other K-means-models can be found in Appendix C.

### 3.4 Metrics

For the single-step retrosynthesis model evaluation we computed four different metrics. The first one is the top*n* accuracy, which is over-evaluated for this kind of task for the reasons we have explained before. In this section, we explain in more detail the other three metrics considered.^6,32^

#### 3.4.1 Round-trip accuracy

The round-trip accuracy metric, unlike the top*n* accuracy, takes the top*k* predictions for a molecule and applies on top of them a forward prediction model (see Section 3.2). If the original molecule is recovered through the forward model, then that reaction contributes positively to the accuracy. This is also how we define a prediction to be valid. More specifically, given *X* = {(*x*_*i*_,*y*_*i*_)…(*x*_*N*_,*y*_*N*_)} our dataset of *N* target products with target precursors, we define the top*k* round-trip accuracy (RT_*k*_) as follows:1
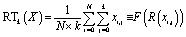
where *x*_*i*,*j*_ is the *j*th prediction for the *i*th sample, *R* is the retrosynthesis model and *F* is the forward translation model.

#### 3.4.2 Class diversity

Class diversity is the most interesting metric in our analysis. It measures the average number of reaction classes predicted for each target product molecule. For example, considering the first *k* predictions for a set of test molecules, a class diversity of 5 means that, on average, the (valid) precursors predictions belong to five different reaction classes. Here “valid” is in the sense of round-trip accuracy. Again, in mathematical terms, given *X* = {(*x*_*i*_,*y*_*i*_)…(*x*_*N*_,*y*_*N*_)} our dataset of *N* target products with target precursors, we define the top*k* class diversity (CD_*k*_) as follows:2
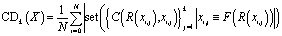
where set() is the set operation on the elements and ‖ is the set cardinality. As above, *x*_*i*,*j*_ is the *j*th prediction for the *i*th sample, *R* is the retrosynthesis model and *F* is the forward translation model. *C* is the classification model used to predict the classes (see Section 3.2).

#### 3.4.3 Coverage

The coverage is the fraction of test samples for which there exists at least one valid prediction (in the round-trip accuracy sense). Given *X* = {(*x*_*i*_,*y*_*i*_)…(*x*_*N*_,*y*_*N*_)}, our dataset of *N* target products with target precursors, we define the top*k* coverage (CV_*k*_) as follows:3
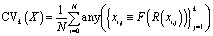
where any() outputs 1 if at least one valid prediction exists among the top*k* for that sample.

## 4 Conclusions and outlook

Exploration and diversity are at the heart of any application of language models to retrosynthesis algorithms. Current retrosynthesis models focus mainly on predicting the reported ground truth, and do not take into account the ability to generate alternatives. Our work is the first AI approach tackling and analysing retrosynthetic diversity directly. We have presented a cluster token prompt-based model that effectively increases diversity in predictions for single-step retrosynthesis. In addition to improving on other measures, our approach can increase class variety by a factor of two or more over the baseline. The decreased validity of the disconnections is softened by the nature of the prompts which act as ‘soft-conditioning’ terms as opposed to the valid/invalid application of reaction templates. Higher diversity comes at the cost of a drop of around 15 percentage points in the top20 round-trip accuracy. However, round-trip accuracy is still a noisy metric that depends on the applicability domain of the forward reaction prediction model and does not take duplicates into account and as such cannot be fully trusted. In terms of computation time, the prompting approach increases the generation of the predictions by a factor proportional to the number of cluster tokens used. Incorporating a diversity-boosted single-step retrosynthesis model, into a multi-step pipeline (for example, Beam Search) to recursively build disconnection trees, offers a set of very diverse reactions from which to choose. This strategy improves the search for less obvious and more engaging paths. It becomes even more of interest in an interactive framework where chemists can be assisted by AI to plan their retrosynthetic route relying on a wide variety of chemical disconnection recommendations and indirectly less bias.

## 5 Appendix

### A Open-source dataset results

The procedure applied to the proprietary Pistachio dataset^21^ in the main manuscript was also applied to an open-source dataset, USPTO 50k,^26^ for reproducibility reasons. This dataset was chosen because it is the only open-source dataset with public chemical reactions classification, performed by Schneider *et al.*^35^

The whole data processing procedure, the dataset, the scripts and the models are available with the code. For this smaller dataset, we built three random models and three models based on clustering of reaction fingerprints. We used 2, 5 and 10 tokens for the clustering. As for Pistachio, we chose the best cluster token prompt-based models by comparing them against the validation set. We concluded the analysis with the confrontation against the baseline on five random seeds on the test set.

**Fig. 8:**
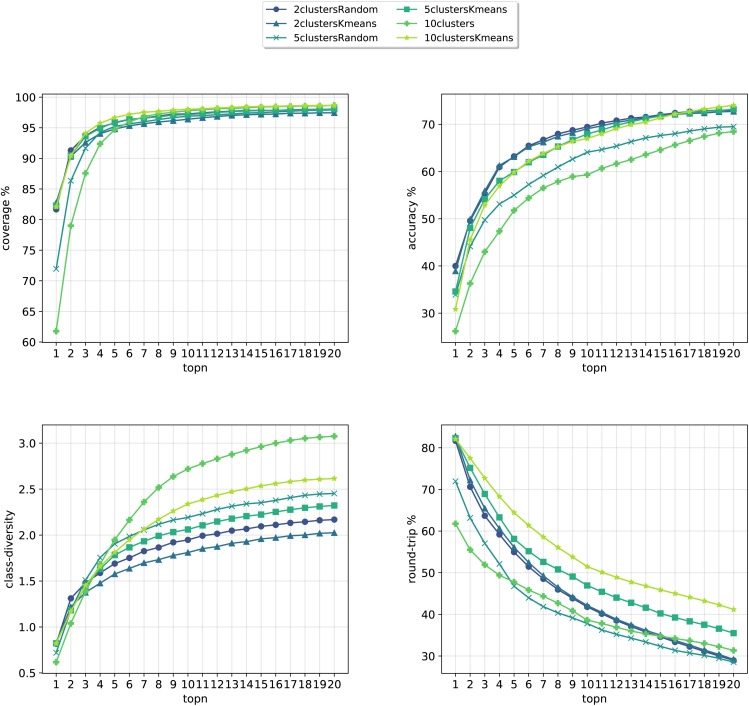
Metrics for the models trained on USPTO. Top left: coverage. Top right: top*n* accuracy. Bottom left: class diversity. Bottom right: round-trip accuracy.

**Fig. 9:**
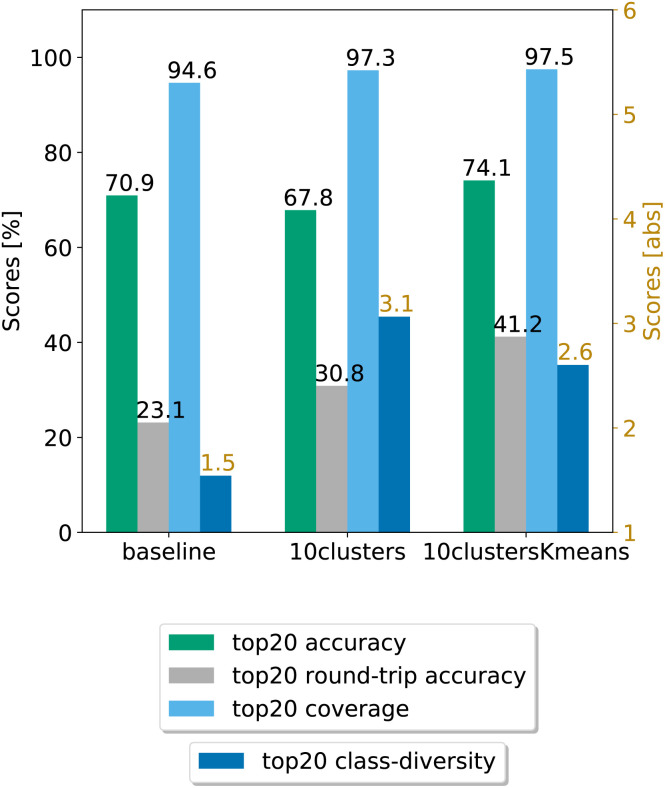
Final comparison of the best cluster token prompt-based models and the baseline against the test set for the open source dataset. The values of the metrics reported are averaged across 5 random seeds. For convenience, standard error values are reported in Table 2.

In Fig. 8, we compare the cluster token prompt-based models trained on USPTO 50k, while in Fig. 9, we compare the final best models.

Differently from the results with Pistachio we notice that the models can better predict the ground truth precursors. It is to be noted that USPTO 50k is a smaller dataset where only reactants and not reagents are reported (differently from Pistachio), so the training task is much easier than with Pistachio. At the same time, though, the round-trip accuracy has a quite low value, even if the forward model for the evaluations was trained with the same USPTO 50k dataset and reached an Accuray of 77.46% (and 95.29% accuracy on the classification model). This behaviour can be ascribed to the fact that the dataset is too small and it is not able to generalize sufficiently well. On top of this, it is to be noted that for USPTO-50k the accuracy and round-trip accuracy of the prompt models increase a lot compared to the baseline model. This trend is inverted for the case of the Pistachio dataset (see Fig. 4). We believe that the increased accuracy with respect to the baseline can be ascribed to the size and easiness of the open-source dataset. Indeed, a model trained on USPTO 50k sees less examples from each of the classes and this gives more specificity to the conditioning token, which gives an additional hint to the model for the prediction with respect to the baseline (higher top*N* accuracy). Then, being the task easier (only reactants), the round-trip accuracy shows also an increase. For Pistachio this does not happen because the reaction space is much larger and diverse and includes reagents. The conditioning in this case has access to more reactions and therefore many predictions can include the original disconnection with different reagents (lower top*N* accuracy), which the proxy model might not be confident enough to validate (lower round-trip accuracy).

Looking at Fig. 9, we see that for the 10clusters model, corresponding to using all the reaction classes ids as single tokens, the class diversity increases to 3.1. The best top20 accuracy as well as the round-trip accuracy is reached by the 10clustersKmeans model.

We also report the standard error values at top20 predictions for the best models, computed with the same random seeds. The values can be found in Table 2. We observe that the error bar is more significant for the open-source models. This can be ascribed to the smaller dataset. Indeed, for only 50k data points we cannot create sufficiently general splits as for the 2 million data samples from Pistachio. The 10clustersKmeans model is the best compromise through all metrics.

**Table 2:** Comparison of the cluster token prompt-based models for USPTO 50k against the baseline on the test set. Uncertainty bounds are computed based on the standard error and reported in the table

Model	Coverage	Accuracy	Round-trip accuracy	Class diversity
Baseline	94.64 ± 0.97%	70.94 ± 0.31%	23.13 ± 0.46%	1.54 ± 0.29
10clusters	97.28 ± 0.10%	67.84 ± 0.47%	30.84 ± 0.65%	**3.06 ± 0.07**
10clustersKmeans	**97.49 ± 0.15%**	**74.09 ± 0.17%**	**41.21 ± 0.69%**	2.60 ± 0.04

### B Baseline and other plots

**Fig. 10:**
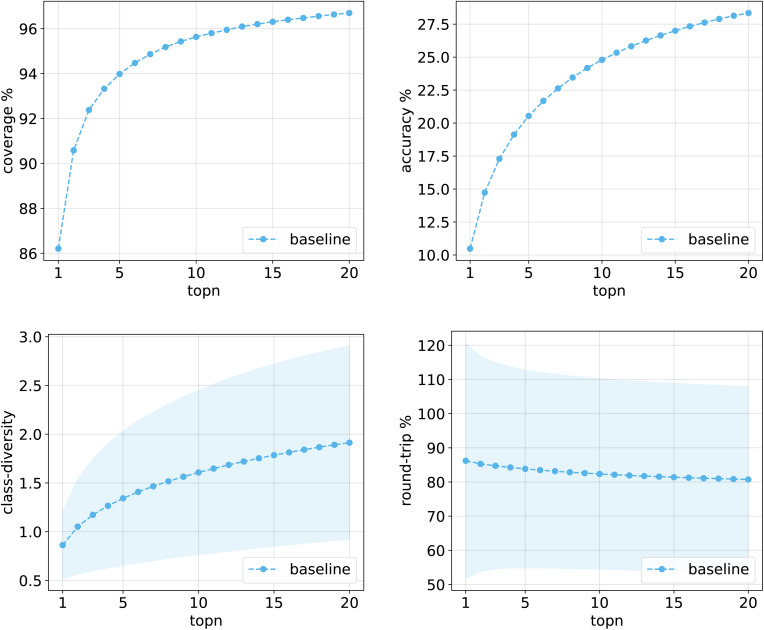
Metrics for the baseline model trained on Pistachio. Top left: coverage. Top right: top*n* accuracy. Bottom left: class diversity. Bottom right: round-trip accuracy.

Fig. 10 shows the values for the metrics of interest for the baseline model. The shades mark the standard error bounds for class diversity and round-trip accuracy.

**Fig. 11:**
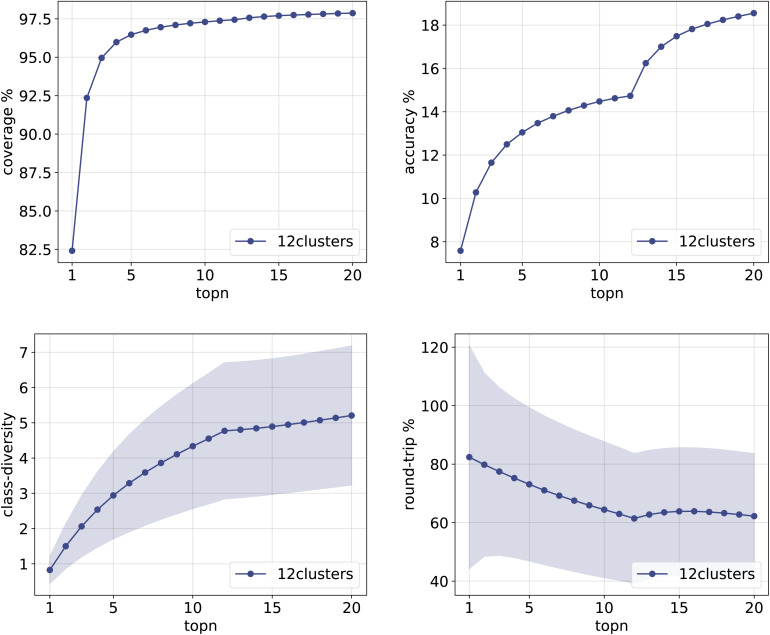
Metrics for the 12clusters model trained on Pistachio. Top left: coverage. Top right: top*n* accuracy. Bottom left: class diversity. Bottom right: round-trip accuracy.

**Fig. 12:**
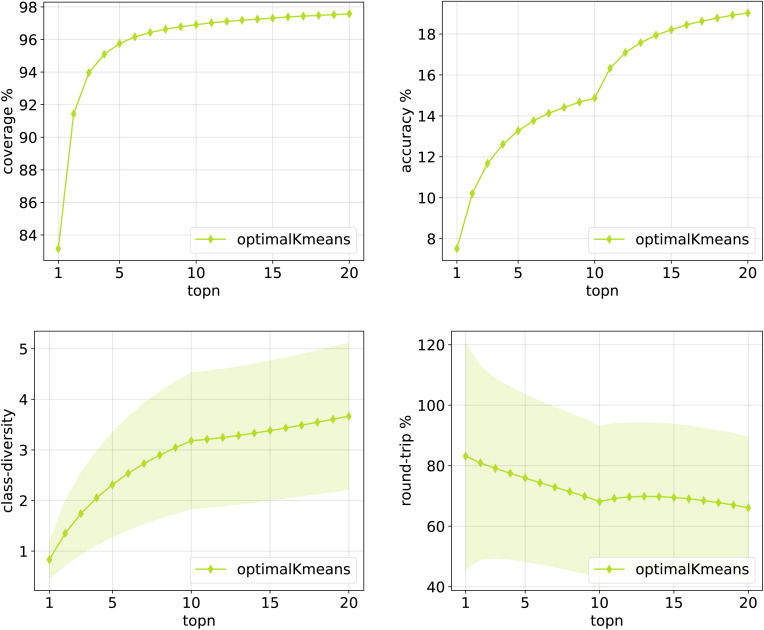
Metrics for the optimalKmeans model trained on Pistachio. Top left: coverage. Top right: top*n* accuracy. Bottom left: class diversity. Bottom right: round-trip accuracy.

The same plots are reported in Fig. 11 and 12 for the 12clusters and the optimalKmeans models. For all models, it can be observed that the standard error on the class diversity is quite high, changing a lot across compounds, but it is the same for the cluster token prompt-based models and the baseline.

### C Kmeans plots

**Fig. 13:**
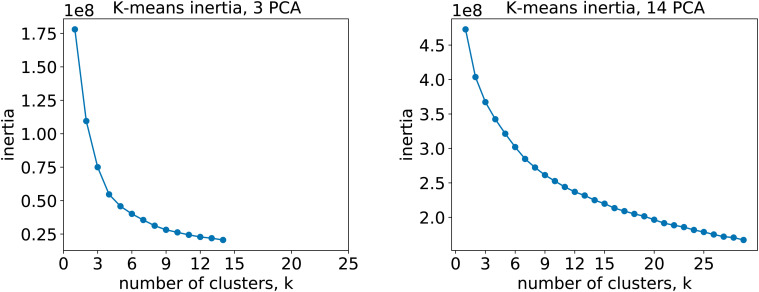
Inertia plots for the K-means clustering. Different K-means algorithms with increasing number of clusters were run on both the 3-components fingerprints (left) and the 14-components fingerprints (right).

**Fig. 14:**
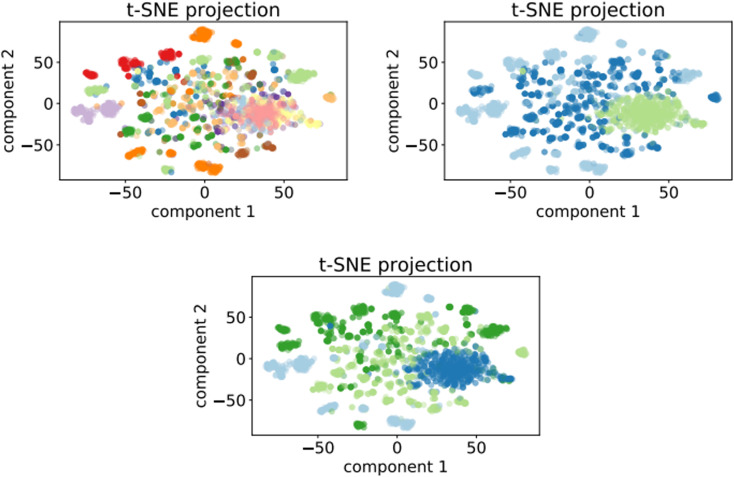
Top left: t-SNE projection for 50 000 samples of the 12clustersKmeans model. Top right: t-SNE projection for 50 000 samples of the 3clustersKmeans model. Bottom: t-SNE projection for 50 000 samples of the 4clustersKmeans model.

In this section we report the inertia plots for the K-means algorithm (Fig. 13), as well as the clustering plots for all the prompt-based K-means models (Fig. 14). The clustering plot for the optimalKmeans model can be found in Fig. 7.

## Ethics statement

This material is the authors' own original work, which has not been previously published elsewhere. The manuscript is not currently being considered for publication elsewhere and it reflects the authors' own research and analysis in a truthful and complete manner.

## Data and code availability

The code used to train the high diversity models can be found at https://github.com/rxn4chemistry/rxn_cluster_token_prompt. Moreover, we provide the cleaned open-source dataset on which it is possible to reproduce the procedure, as well as the models trained on USPTO (details in the GitHub repository). Results for the open-source dataset are reported in Appendix A.

The cluster token prompt models trained with Pistachio are also accessible through the IBM RXN for Chemistry website.^34^

## Author contributions

A. Toniato conceptualized the method and performed the experiments. All authors contributed to the formal analysis of the results and manuscript draft. T. Laino supervised the work.

## Conflicts of interest

There are no conflicts to declare.

## Supplementary Material
